# Forkhead Box Protein O3 Transcription Factor Negatively Regulates Autophagy in Human Cancer Cells by Inhibiting Forkhead Box Protein O1 Expression and Cytosolic Accumulation

**DOI:** 10.1371/journal.pone.0115087

**Published:** 2014-12-29

**Authors:** Wan Long Zhu, Honglian Tong, Jing Tsong Teh, Mei Wang

**Affiliations:** 1 Program in Cancer and Stem Cell Biology, Duke-NUS Graduate Medical School, Singapore, Singapore; 2 Department of Biochemistry, National University of Singapore, Singapore, Singapore; Peking University Health Science Center, China

## Abstract

FoxO proteins are important regulators in cellular metabolism and are recognized to be nodes in multiple signaling pathways, most notably those involving PI3K/AKT and mTOR. FoxO proteins primarily function as transcription factors, but recent study suggests that cytosolic FoxO1 participates in the regulation of autophagy. In the current study, we find that cytosolic FoxO1 indeed stimulates cellular autophagy in multiple cancer cell lines, and that it regulates not only basal autophagy but also that induced by rapamycin and that in response to nutrient deprivation. These findings illustrate the importance of FoxO1 in cell metabolism regulation independent of its transcription factor function. In contrast to FoxO1, we find the closely related FoxO3a is a negative regulator of autophagy in multiple cancer cell lines, a previously unrecognized function for this protein, different from its function in benign fibroblast and muscle cells. The induction of autophagy by the knockdown of FoxO3a was found not to be mediated through the suppression of mTORC1 signaling; rather, the regulatory role of FoxO3a on autophagy was determined to be through its ability to transcriptionally suppress FoxO1. This complicated interplay of FoxO1 and FoxO3a suggests a complex checks- and balances-relationship between FoxO3a and FoxO1 in regulating autophagy and cell metabolism.

## Introduction

Autophagy is a highly conserved cellular process, central to the response of cell to nutrition/energy as well as growth factor status [1], [2]. Appropriately, one of the major upstream regulators of autophagy is PI3K-AKT-mTOR signaling, sensors for growth factor stimulation, amino acid and cell energy levels that are central to cell growth and proliferation [3]–[5]. Indeed, autophagy is regulated in parallel with cellular metabolism and proliferation, forming an integrated response to the external and internal environments. For example, when nutrient and energy levels are perceived as low, cell proliferation and anabolic activity decrease while autophagy increases to provide energy and macromolecules for essential cellular functions [6]. While inhibition of autophagy can result in cell death, prolonged induction of excessive catabolic activity, such as autophagy, can also lead to cell demise; both of these processes can be exploited as new approaches for cancer treatment [7]–[10]. Hence, a thorough understanding of autophagy regulation in different cell contexts is important in establishing the potential for therapeutic manipulation of this process.

Forkhead box protein O transcription factors (FoxOs) are evolutionarily conserved proteins that occupy regulatory nodes in multiple signaling pathways important for the cellular response to external energy, nutrition, and growth factor stimulations. As such, they are involved in regulating anabolic and catabolic states of cells, and in growth, proliferation, and cell death decisions [11]–[17]. It is not surprising, therefore, that the dysfunction of these proteins impacts on pathological processes such as diabetes, aging and cancer [12], [16]–[19].

FoxO proteins have been reported to be regulators of cellular autophagy, a process that is intimately pegged to the anabolic/catabolic state of the cell. Multiple studies have suggested that FoxO3a in particular promotes the expression of autophagy genes, leading to increased autophagy [20]–[22]. These and other findings have led to the notion that FoxO proteins in general are activators of autophagy through their function as transcription factors [23], [24]. In this view, the functions of different FoxO proteins are considered similar and overlapping with regard to the promotion of autophagy, with tissue distribution accounting for their differential impact in specific cell contexts. One important focus of the regulation of FoxO proteins has been on their cellular localization, which is reversibly regulated by their post-translational modifications, primarily that of phosphorylation [25]–[28], and acetylation [29], [30] in response to environmental stimuli. These post-translational modifications are intimately connected to the cellular localization of FoxO proteins and their interactions with effectors, and therefore are considered to be important in regulating the level of activities of these proteins [31], [32]. Indeed, recent findings have suggested that cytosolic FoxO1 can promote autophagy, in response to nutritional stress, by direct interaction with Atg7, demonstrating the complicated roles of this group of proteins in regulating autophagy [33].

It was recently reported that FoxO3a can promote FoxO1-dependent autophagy in human embryonic kidney and mouse embryonic fibroblast cells, which is mediated by FoxO3a up-regulation of PI3K catalytic subunit, subsequent AKT activation and increased cytosolic distribution of FoxO1 [34]. In contrast, we found that FoxO3a inhibits, rather than enhances, autophagy in multiple cancer cell lines. Further, FoxO3a suppression of autophagy appears to be mediated by down-regulating the transcription of FoxO1, providing new insight into the ways FoxO3a and FoxO1 can interact and exert opposing effects on cellular autophagy. These findings have revealed an unexpected role of FoxO3a in autophagy, and highlight the complexity of FoxO signaling and its biological impact in different cell contexts.

## Materials and Methods

### Reagents and antibodies

Antibodies recognizing human GAPDH, FoxO1 (C29H4), FoxO3a (75D8), p-4EBP1(T37/46), p-S6 (S240/244), Atg5, Flag, and Histone H3 were from Cell Signaling Technology (Danvers, MA); Antibodies for LC3 (APG8A) was from Abgent (San Diego, CA). The protease inhibitor cocktail was from Roche (Basel, Switzerland). All cell lines used in the study were obtained originally from American Type Culture Collection.

### Cell culture and drug treatment

Cells were maintained at 37°C with 5% CO_2_ in DMEM (Invitrogen, North Andover, MA) supplemented with 10% FBS (Hyclone, Novato, CA), 50 units/ml penicillin and 50 µg/ml streptomycin (Invitrogen). Additional treatment with different inhibitors, such as rapamycin (Sigma-Aldrich, St. Louis, MO), cycloheximide (Sigma-Aldrich), and chloroquine (Sigma-Aldrich) were carried out in DMEM supplemented with 10% FBS; details of each treatment condition are noted in the respective figure legend. For different starvation treatment, cells were culture in serum free, or D-glucose free medium, respectively.

### siRNAs, primers and plasmids

siRNA-1 of FoxO1 (5′- UUG UAC AGG UGU CUU CAC UUG GGU C-3′), siRNA-1 of FoxO3a (5′-AUU GAC CAA ACU UCC CUG GUU AGG C-3′) and siRNA of Atg5 (5′-AUU CCA UGA GUU UCC GAU UGA UGG C-3′) were purchased from Invitrogen; siRNA-2 of FoxO1 (5′-ACA UGC UCA GCA GAC AUC UGC AGU U-3′) [35] and FoxO3a (5′-GAG CUC UUG GUG GAU CAU C-3′) [36] was synthesized from Sigma-Aldrich. Forward and reverse primers for Real-time PCR were: FoxO1, 5′-AACCTGGCATTACAGTTGGCC-3′ and 5′-AAATGCAGGAGGCATGACTACGT-3′; FoxO3a, 5′-CTTCAAGGATAAGGGCGACAG-3′ and 5′-TCGTCCTGGACTTCATCCA AC-3′; Atg4c, 5′-GCAGGAGATTGGTATGGACCAGCT-3′ and 5′-GGATGCCTT GCTTCTTCAACTGCT-3′; LC3, 5′-AGACCTTCAAGCAGCGCCG-3′ and 5′-ACACTGACAATTTCATCCCG-3′; Atg7, 5′-GATCCGGGGATTTCTTTCACG-3′ and 5′-CAGCAGCTTGGGTTTCTTGAT-3′; Atg12, 5′-TCTATGAGTGTTTTGGCAGTG-3′ and 5′-ATCACATCTGTTAAGTCTCTTGC-3′; Bnip3, 5′-GCCCGGGATGCAGGAGGAGA-3′ and 5′-GAGCAGCAGAGATGGAAGGAAAAC-3′; 18S, 5′-AAGTTCGACCGTCTTC TCAGC-3′ and 5′-GTTGATTAAGTCCCTGCCCTTTG-3′. Flag-FoxO1 [37] and Flag-FoxO3a [38] expression plasmids were purchased from Addgene (Cambridge, MA). Flag-FoxO1-ΔDB (deletion of residues 208–220 of FoxO1) and Flag-FoxO3a-3A (Alanine substitution of Thr 32, Ser 253 and Ser 315 of FoxO3a) expression plasmids were constructed using QuikChange II Site-Directed Mutagenesis Kit (Stratagene, Santa Clara, CA). Flag-FoxO3a(r), which is wide type FoxO3a protein coded by cDNA resistant to siFoxO3a we used in the study, was generated by mutating eight siRNA-1 of FoxO3a targeting base-pairs in the wild-type Flag-FoxO3a plasmid using QuikChange II Site-Directed Mutagenesis Kit (Stratagene). All plasmids were verified by DNA sequencing. The tandem fluorescent mRFPGFP-LC3 plasmid was a gift from Dr. Tamotsu Yoshimori [39].

### Transfection with siRNA or expression vectors

Transfections were done using Lipofectamine 2000 (Invitrogen) based on manufacturer's protocol; the transfected cells were subjected to desired treatments for 24–48 h after transfection as described in respective figure legends.

### Cytosol-nucleus fractionation

Harvested cells were suspended in cold buffer (100 mM Tris-HCl pH 7.6, 15 mM Mg(OAc)_2_, 10 mM KOAc, 10 mM PMSF, 0.5% NP40, 1% protease inhibitor cocktail) and left on ice for 20 min, before centrifugation at 2,500 *g* for 5 min at 4°C. The supernatant fraction was collected as the cytoplasmic extract. The pellet was washed once with the same buffer before being suspended at 4°C in nuclei lyses buffer (100 mM Tris-HCl pH 7.6, 42 mM NaCl, 50 mM EDTA, 10 mM PMSF, 2% SDS, 1% protease inhibitor cocktail). Following 10 min incubation on ice, the suspension was sonicated for 3 min on ice before centrifugation at 12,000 *g* for 15 min at 4°C. The supernatant fraction was collected as the nuclear extract.

### Immunoblotting and analysis

Cells subjected to the treatment indicated in the appropriate figure legend were harvested, lysed, and protein concentration determined according to standard protocol. Proteins were separated by SDS-PAGE, and immunoblot analysis was performed using an enhanced chemiluminescence procedure (GE Healthcare, Piscataway, NJ).

### Confocal imaging and analysis

Cells were fixed with 4% paraformaldehyde, followed by permeabilization with cold methanol. For cells went through immunofluorescent labeling, the fixed, permeabilized cells were incubated with blocking buffer (5% normal goat serum, 0.3% Triton X-100 in PBS) for 1 h at room temp, followed by incubation in the appropriately diluted antibody in antibody dilution buffer (1% BSA, 0.3% Triton X-100 in PBS) overnight at 4°C. Cells were then washed three times with PBS and subsequently incubated with either FITC-goat anti-rabbit (1∶1000) or Rhodamine-goat anti-mouse IgG secondary antibodies (1∶1000), as appropriate, in antibody dilution buffer for 1 h at room temp. All confocal images were taken with a Carl Zeiss LSM 710 confocal microscope (Oberkochen, Germany). Imaging data were analyzed by Metamorph Analysis Software (Molecular Devices Inc., Sunnyvale, CA). Colocalization efficiency of mRFP to that of GFP fluorescent signals was measured using ImageJ software.

### Quantitative PCR assay

Cells subjected to the siRNA knockdown or gene over-expression protocols were harvested according to standard protocol. Total RNA was extracted using RNeasy Mini Kit (QIAGEN, Hilden, Germany) according to the manufacturer's instructions. Subsequently, first-strand cDNA was synthesized under standard conditions with the Superscript First-strand Synthesis System (Invitrogen). Quantitative PCR was carried out with CFX96 Real-Time System (Bio-Rad, Hercules, CA). The quantity of the transcript was normalized to the level of ribosomal proteins 18S.

### Data analysis

Statistical differences were assessed by Student's *t* test. Differences were considered statistically significant at p<0.05(*) and *p*<0.01(**).

## Results

### FoxO3a negatively regulates autophagy, in contrast to FoxO1 which promotes autophagy

**Figure 1:**
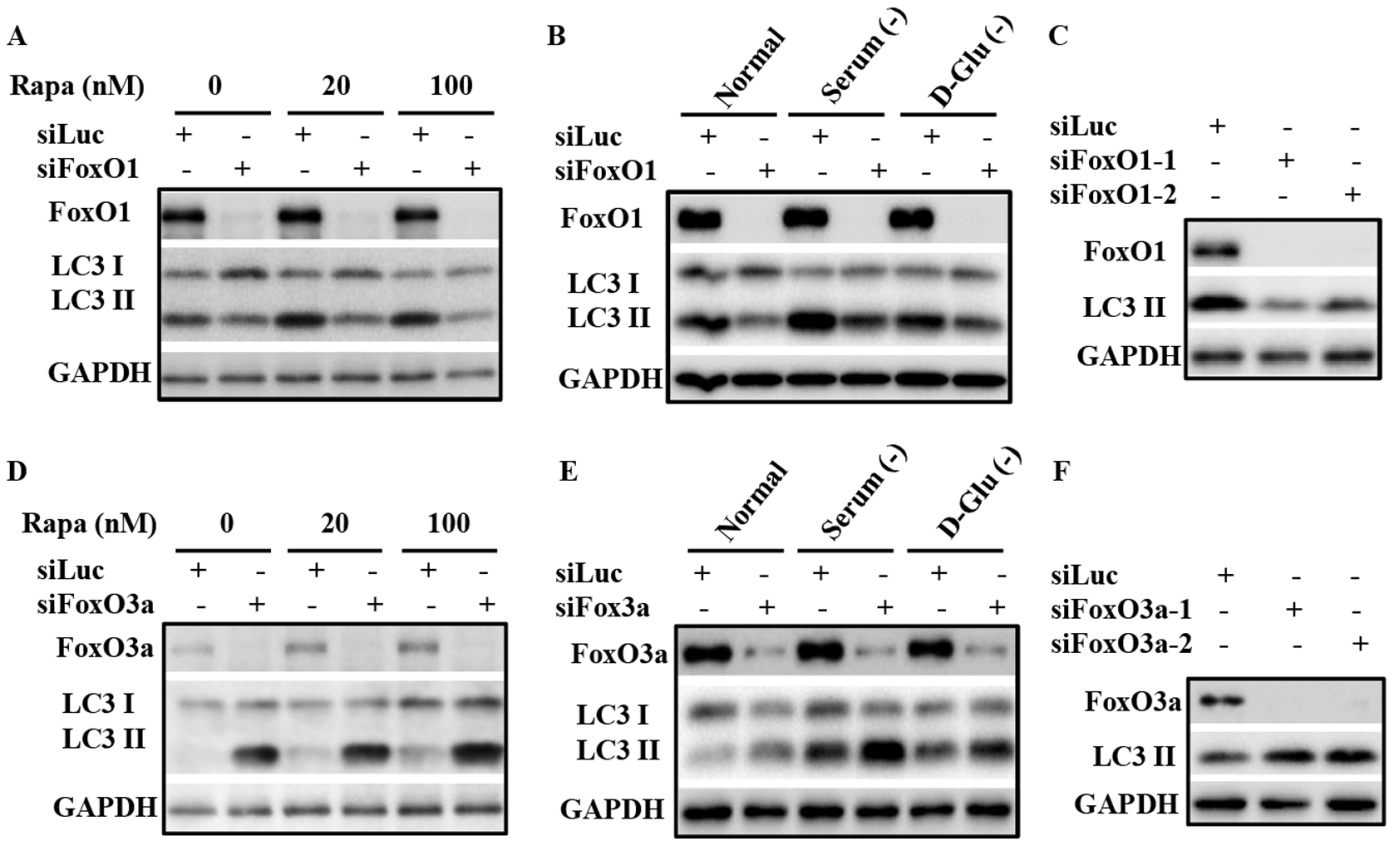
FoxO3a knockdown induces, while FoxO1 knockdown inhibits, autophagy. (A) Immunoblot analysis of lysates from PC3 cells transfected with control siRNA (siLuc) or that targeting FoxO1 (siFoxO1), with or without rapamycin treatment. PC3 cells were transfected with the indicated siRNA for 48 h before subsequent treatment with DMSO, 20 nM or 100 nm rapamycin (Rap) for 24 h prior to harvest and processing. (B) PC3 cells were transfected with the indicated siRNA for 48 h prior to media change, whereupon the cells were subjected to the indicated growth conditions; these conditions are DMEM in the presence (Normal) or absence of 10% FBS (serum -), or in the absence of D-glucose (with 10% FBS), as indicated. Cells were harvested 6 h after exposure to these conditions, and processed for immunoblot analysis of the indicate proteins. (C) Knockdown of FoxO1 with two targeting siRNAs suppresses autophagy in PC3 cells. (D, E, F) illustrate similar study procedures as (A, B, C), but with siRNAs targeting FoxO3a in PC3 cells. All experiments have been performed three times with similar results.

**Figure 2:**
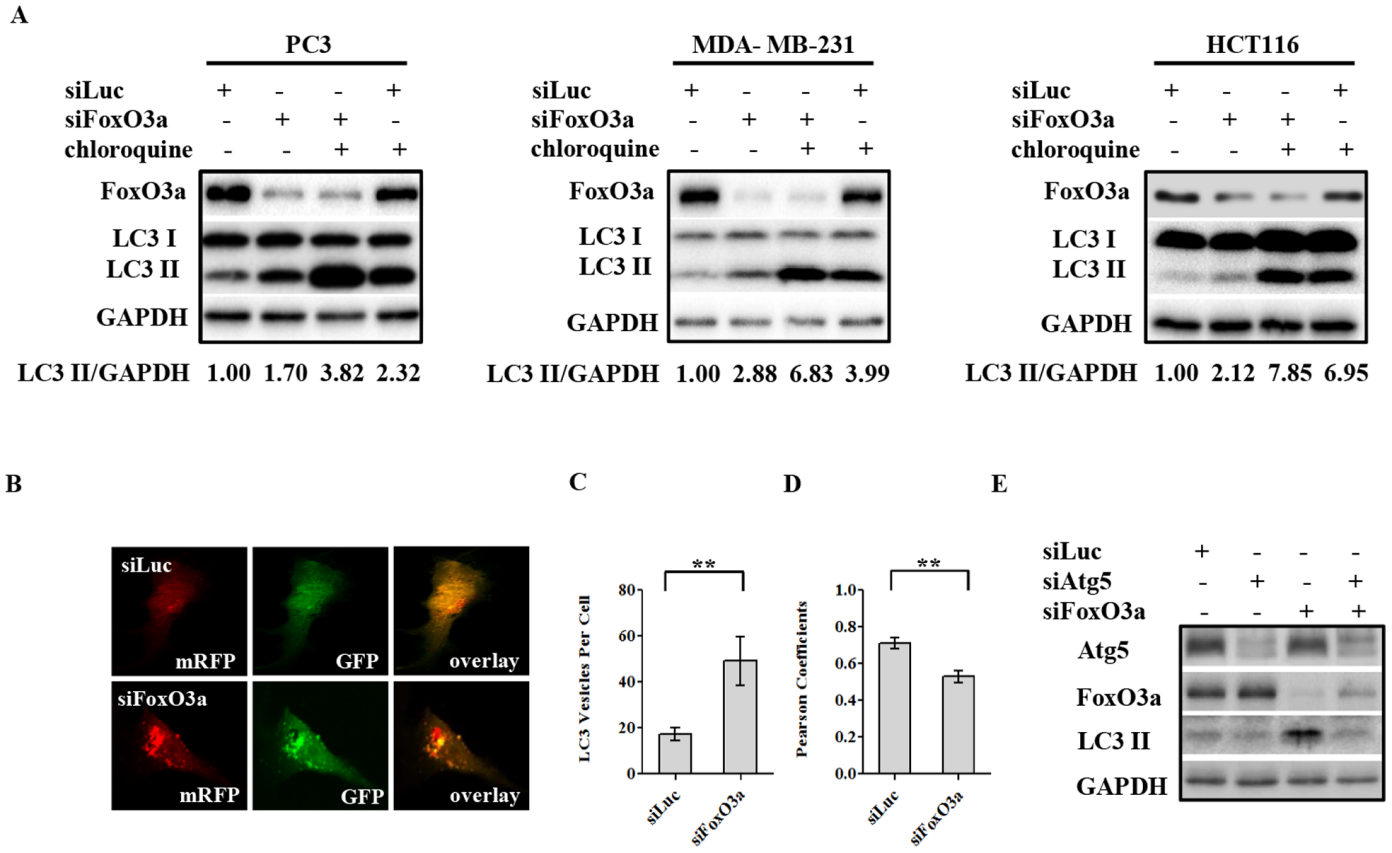
FoxO3a knockdown increases autophagic flux. (A) PC3, MDA-MB-231, and HCT116 cells were transfected with control siRNA (siLuc) or that targeting FoxO3a (siFoxO3a). Subsequently, these cells were exposed to control vehicle or 50 µM chloroquine 72 h post transfection for 3 h before cell lysates preparation for immunoblot analysis of the indicated proteins. (B) Confocal microscopy of MDA-MB231 cell stably expressing tandem fluorescent mRFP-GFP-LC3 following transfection by either control or FoxO3a siRNA. Images were taken 72 h after transfection of the indicated siRNA. (C) Quantitative analysis of the images from the experiment shown in panel B using MetaMorph software to determine the average number of RFP-positive particles per cell in control (siLuc) and siFoxO3a treated cells. (D) Colocalization analysis of RFP and GFP from experiment described in panel B to assess autophagic progression. RFP and GFP colocalization indicated by Pearson Coefficients was analyzed by ImageJ software. In both (C) and (D), >50 cells were analyzed for each condition. Data are presented as Mean ± S.E.M. (“**”, *p*<0.01), and detailed methods are described in Experimental Procedures. (E) PC3 cells were transfected with control siRNA (siLuc) or that targeting Atg5 (siAtg5), FoxO3a (siFoxO3a), or combination of both, as indicated. Cells were harvested 72 h after transfection and the lysates were processed for analysis.

Several reports have demonstrated the involvement of FoxO proteins in regulating autophagy, but the precise manner of this regulation and the roles different FoxO proteins may play in this process, particularly the roles of different FoxO proteins in different cell context, requires further clarification. Suppression of FoxO1 levels in prostate cancer PC3 cells by siRNA knockdown inhibited cellular autophagy, both under basal conditions and that induced by rapamycin (Fig. 1A) or serum and glucose deprivation (Fig. 1B). Two siRNAs targeting FoxO1 suppressed autophagy in PC3 cells, as assessed by LC3-II levels, suggesting a target specific effect of this regulation (Fig. 1C). These data are consistent with the notion that FoxO1 is a positive regulator of autophagy [23], [33], [34]. To investigate the role of FoxO3a in PC3 cells, similar studies were performed using siRNA targeting FoxO3a. Surprisingly, an opposite effect was observed upon suppression of FoxO3a expression; siRNAs targeting FoxO3a enhanced basal autophagy as well as that induced by rapamycin, measured by lipidated LC3 levels (Fig. 1D). This reduction of FoxO3a expression further enhanced autophagy induced by either serum or glucose starvation (Fig. 1E), suggesting a general involvement of FoxO3a in the regulation of autophagy in response to nutrition and growth signals in PC3 cancer cells. The induction of autophagy by FoxO3a suppression was validated with two FoxO3a targeting siRNAs (Fig. 1F), diminishing the likelihood of this being an off-target effect. Previous studies in myotubes and fibroblasts have shown that FoxO3a plays important roles in promoting autophagy [20], [34]; therefore this result suggests complex and cell context-specific roles of FoxO3a in regulation of autophagy. It seemed likely that there are distinct differences between muscle and fibroblast cells and epithelial-derived cancer cells in this regulation. To determine whether the surprising negative regulation of autophagy by FoxO3a was limited to PC3 prostate cancer cells, we examined the impact of FoxO3a silencing in HCT116 colon and MDA-MB-231 breast cancer cell lines. Similar to the observation in PC3 cells, knockdown of FoxO3a led to LC3-II elevation in both cell lines (Fig. 2A), demonstrating this negative regulation of autophagy by FoxO3a is not limited to a single cancer cell line.

The pattern of LC3-I and LC3-II levels alone is not sufficient to illustrate the actual autophagy process, as either increased initiation of autophagy or reduced progression of autophagy to lysosomal degradation system can lead to the elevation of LC3-II levels. To clarify the role of FoxO3a in this process, we determined the impact of FoxO3a silencing on actual autophagy flux using two approaches. One approach involved treatment of the cells with chloroquine in the presence of FoxO3a knockdown, which was performed in all three cancer cell lines, i.e. PC3, MDA-MB-231 and HCT116 cells. Suppression of FoxO3a resulted in the accumulation of LC3-II in these cells; and the addition of chloroquine further increased the LC3-II levels, suggesting that FoxO3a suppression increased LC3-II through autophagy induction (Fig. 2A). The second approach involved the measurement of the progression of autophagosomes into the acidic autophagolysosomes by confocal imaging. MDA-MB-231 cells stably expressing the tandem florescence protein mRFP-GFP-LC3 were transfected with FoxO3a siRNA or control siRNA; subsequently, co-localization of GFP with RFP, an indicator of autophagosome-lysosome fusion and protein degradation, was examined [39]. GFP fluorescence is labile in acidic conditions; therefore the loss of green florescence that is observed as delocalization of RFP from GFP tracks the increase in acidity in autophagosomes as they evolve into autophagolysosomes. In this system, FoxO3a silencing not only led to increased LC3 positive foci, but also to significant reduction in GFP co-localization with RFP, reflecting the status of increased autophagy flux (Fig. 2B, 2C, 2D). Further, siRNA mediated reduction of Atg5 levels inhibited autophagy induced by FoxO3a knockdown (Fig. 2E), providing evidence that suppression of FoxO3a promote autophagy induction in an Atg5 dependent manner. These data clearly establish that, while FoxO1 promotes autophagy as expected, FoxO3a negatively regulates the process in these cancer cells, playing an opposite role.

**Figure 3:**
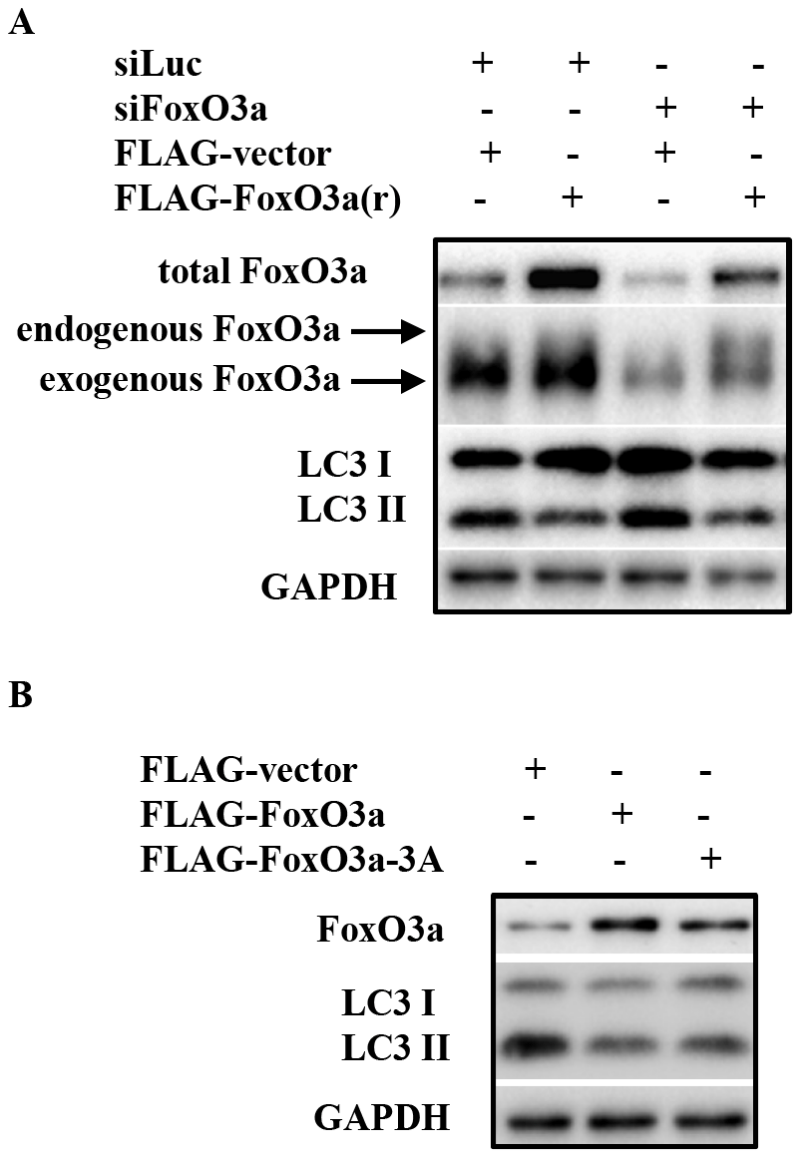
FoxO3a over expression inhibits autophagy. (A) PC3 cells were transfected with either control siRNA or that targeting FoxO3a; each group of cells was also co-transfected with either ectopic expression vector control or that express FoxO3a(r). The cell lysates were harvested for immunoblot 72 h post transfection. (B) PC3 cells were transfected with 3 different expression plasmids, which are vector-Flag control, Flag-FoxO3a or Flag-FoxO3a-3A, respectively; the cell lysates were prepared 72 h post transfection for immunoblot analysis of the indicated proteins.

To further test the hypothesis that FoxO3a is a negative regulator of autophagy in cancer cell lines, a target-specific rescue study was performed in PC3 cells by ectopic expression of FoxO3a(r), which encodes the same protein sequence as wild type FoxO3a but contained silent mutations rendering it resistant to the FoxO3a siRNA. Immunoblot analysis demonstrated that ectopic expression of FoxO3a(r) suppressed both basal autophagy and the autophagy induced by FoxO3a knockdown, in comparison to that in the cells expressing vector control (Fig. 3A). Worth noting, expression of a form of FoxO3a, called FoxO3a-3A (unable to be phosphorylated by AKT), which exclusively localized to the nucleus [20], inhibited the autophagy as much as the wild type FoxO3a that localized both in the nucleus and in cytosol (Fig. 3B). These findings suggested that FoxO3a negatively regulates autophagy through its nuclear, most likely transcriptional function.

### FoxO3a regulation of autophagy is mediated by FoxO1

**Figure 4:**
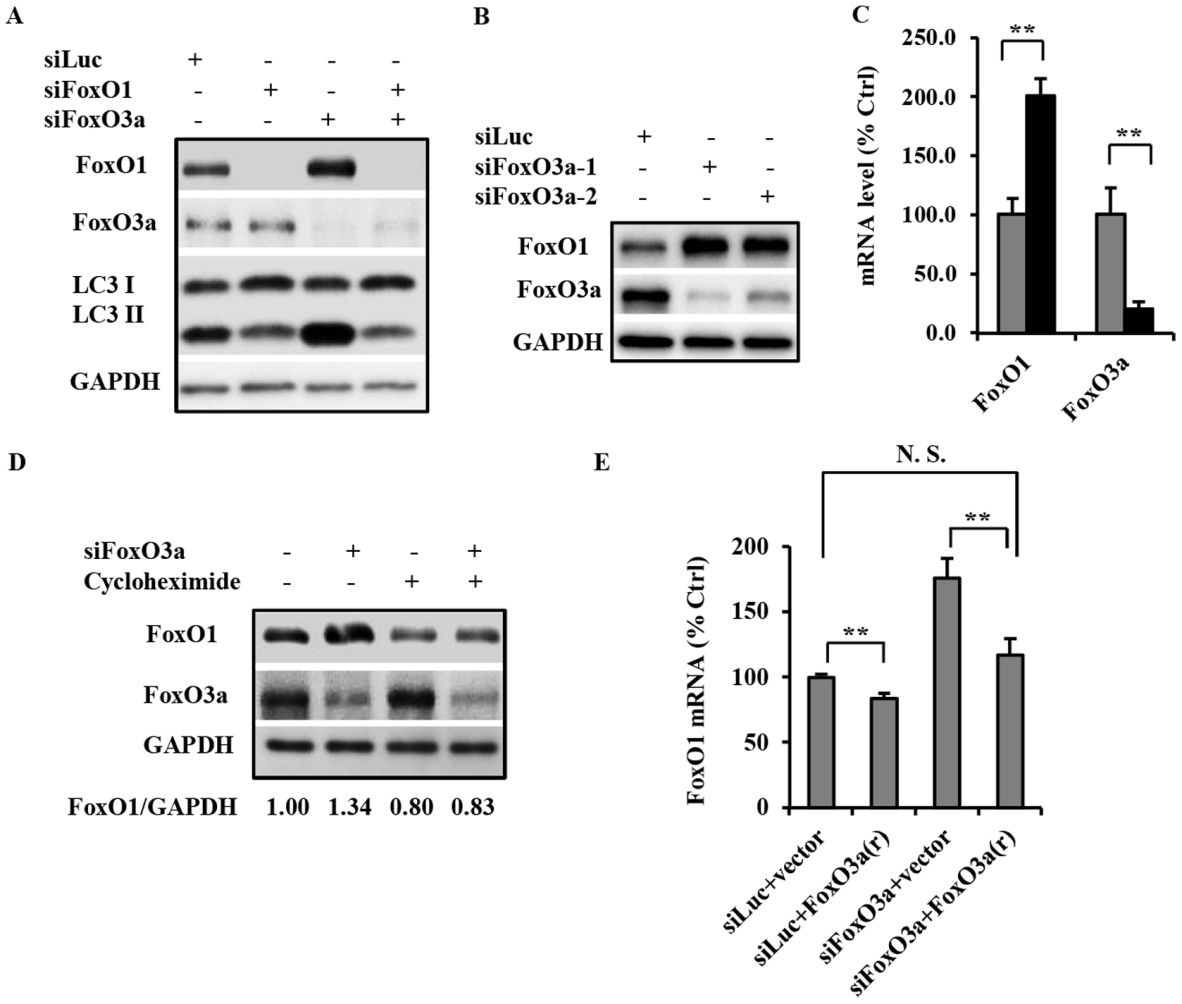
FoxO3a negatively regulates autophagy through inhibition of FoxO1 transcription. (A) PC3 cells were transfected with siRNA for luciferase (siLuc), FoxO1 (siFoxO1), or FoxO3a (siFoxO3a), or combinations, as indicated. Cells were harvested 72 h after transfection, and cell lysates processed for immunoblot analysis of the indicated proteins. (B) FoxO3a knockdown with two FoxO3a targeting siRNA to illustrate the impact on FoxO1 protein level. (C) Real-time PCR analysis of FoxO1 and FoxO3a mRNA levels in control siRNA (gray) or FoxO3a siRNA (black) transfected cells. Cells were harvested and processed 48 h after transfection; 18S ribosomal RNA was analyzed as the normalization control. Data was presented as Mean ± S.D. (“**”, *p*<0.01). (D) PC3 cells were transfected with control siRNA or FoxO3a siRNA as indicated. Following 48 h of transfection, cells were treated with either DMSO control or 10 µg/ml cycloheximide for 24 h as indicated prior to being harvested for immunoblot analysis of the indicated proteins. The FoxO1/GAPDH ratio for each condition is as presented after band quantification by ImageJ. (E) Real-time PCR analysis of endogenous FoxO1 expression level in PC3 cells transfected with either control plasmid (vector) or that expressing FoxO3a(r); in each group of the plasmid transfected cells, either control siRNA or FoxO3a siRNA were concurrently introduced. The cells are harvested for RNA preparation and q-PCR analysis 72 h post transfection; FoxO1 transcript levels were analyzed, as described in Experimental Procedures. 18S was used as the normalization control. Data was presented as Mean ± S.D. (“**”, *p*<0.01). All experiments have been performed three times with similar results.

It was noted that knockdown of FoxO3a resulted in an increase of FoxO1 protein level (Fig. 4A), raising the possibility that FoxO1 mediates the autophagy induction effect from FoxO3a suppression. To investigate the relationship between FoxO1 and FoxO3a in the regulation of autophagy, we concurrently suppressed the expression of these two proteins. Concurrent knockdown of FoxO1 not only inhibited basal autophagy as expected, but also completely abolished the elevation of autophagy induced by FoxO3a knockdown (Fig. 4A), suggesting that FoxO1 is indispensable in autophagy induction resulting from FoxO3a suppression. Transfection of two different siRNAs targeting FoxO3a both resulted in elevated FoxO1 protein level (Fig. 4B), which make the possibility of off-target effects of the siRNAs an unlikely scenario. Consistent to the impact on protein levels, assessment of transcription by real-time PCR showed an increase of FoxO1 expression with FoxO3a knockdown (Fig. 4C), suggesting that FoxO3a transcriptionally regulates FoxO1 level. Furthermore, treatment of cells with cycloheximide concurrently with FoxO3a knockdown eliminated the increase of FoxO1 protein level (Fig. 4D), providing further evidence for FoxO3a regulating the expression, not stability of FoxO1. Taken together, these data support the conclusions that the increase in FoxO1 resulting from FoxO3a knockdown is likely the result of the increased transcription and synthesis of FoxO1.

To further investigate the potential negative transcriptional regulation of FoxO1 by FoxO3a, we assessed the impact of FoxO3a overexpression on endogenous FoxO1 transcription. When introduced into PC3 cells, direct impact of FoxO3a(r) on FoxO1 expression was observed, with or without concomitant knockdown of FoxO3a. The inhibitory effect of FoxO3a(r) on basal FoxO1 expression was modest, but significant. In contrast, introduction of FoxO3a(r) almost completely obliterated the increase of FoxO1 transcription induced by FoxO3a siRNA, bringing it back to near basal level (Fig. 4E). This FoxO3a rescue study provides direct evidence for FoxO3a inhibition of FoxO1 transcription in PC3 cells. Taken together, these results suggest that FoxO3a negatively regulates cellular autophagy by inhibiting the transcription of FoxO1, a positive regulator of autophagy and cell metabolism.

### Elevation in cytosolic FoxO1, resulting from FoxO3a suppression, induces autophagy

**Figure 5:**
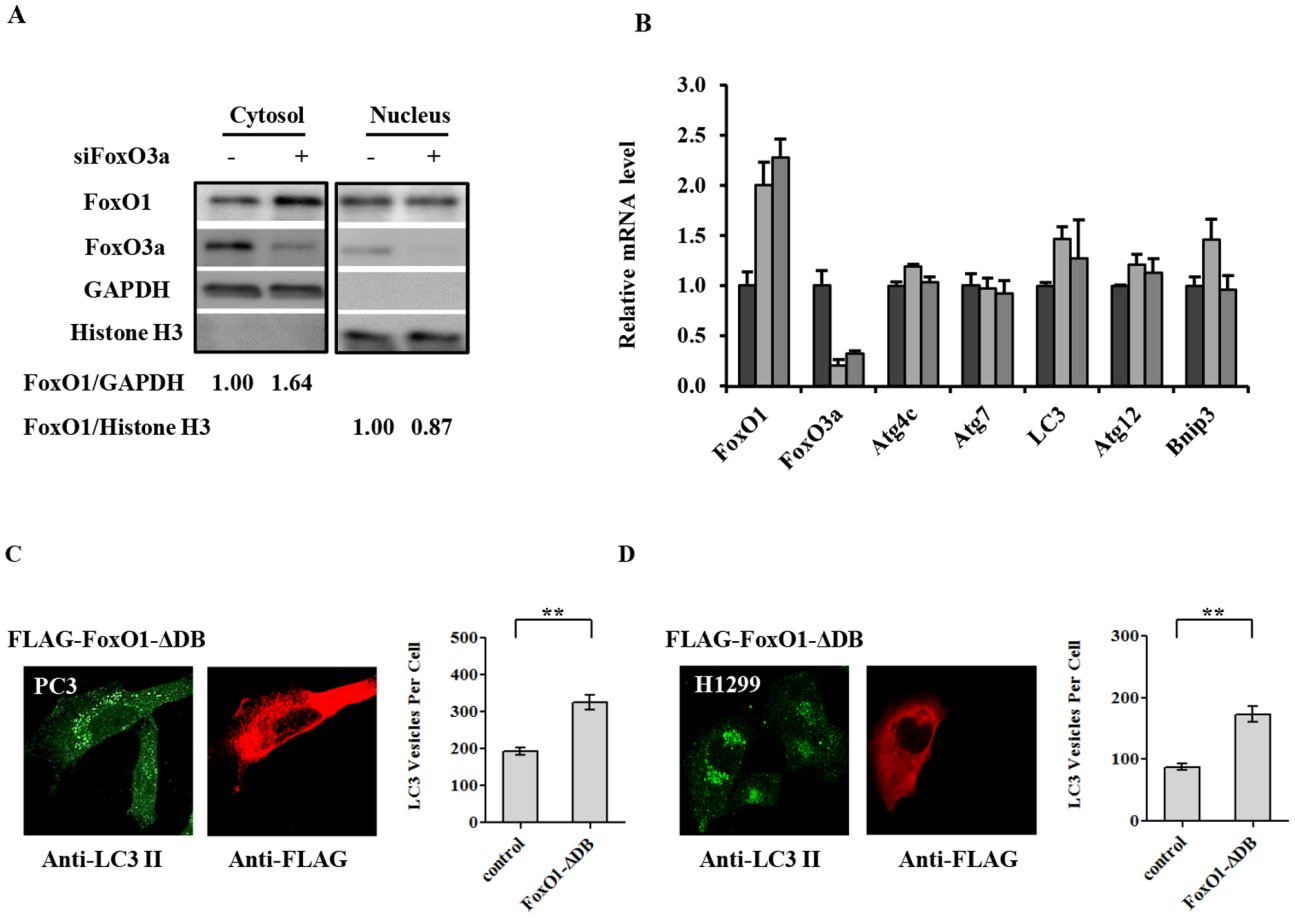
Increased cytosolic FoxO1 resulting from FoxO3a knockdown leads to the elevated level of autophagy. (A) PC3 cells were transfected with siRNA for luciferase (-) or FoxO3a (siFoxO3a) as indicated, and harvested for immunoblot analysis of the indicated proteins 72 h after transfection; see Experimental Procedures for details. Histone H3 and GAPDH were used as loading control for nuclear and cytosolic proteins, respectively. The band quantity ratios, FoxO1/GAPDH and FoxO1/Histone H3, for each condition were obtained using ImageJ. (B) Real-time PCR analysis for the relative expression levels of the indicated autophagy-related genes 48 h after transfection of PC3 cells with control siRNA (black) or two different siRNAs targeting FoxO3a (light and dark grey, respectively). Data was presented as Mean ± S.D. (C, D) Over-expression of a transcription function inactive/cytosolic form of FoxO1, FoxO1-ΔDB, increases autophagy. The quantities of LC3 positive vesicle of both PC3 (panel C) and H1299 (panel D) cells were compared in the same analysis between Flag-FoxO1-ΔDB over-expressing cells and un-transfected cells. FITC and rhodamine tagged secondary antibodies were used for the detection of anti-LC3 or anti-Flag tag, respectively. The autophagy level in each cell population was quantified using MetaMorph software; the data were plotted on the right side of each panel. >50 cells were analyzed for each condition. Data was presented as Mean ± S.E.M. (“**”, *p*<0.01).

It is well-established that FoxO1 positively regulates autophagy by increase the transcription of some autophagy genes [23], [24]. However, recent studies have provided convincing evidence that cytosolic FoxO1 promotes autophagy independent of its transcription regulatory activity [33], [34]. This current study so far has demonstrated that suppression of FoxO3a increased the transcription and total FoxO1protein level, which is the cause of elevated autophagy. It is unclear, however, whether this FoxO1 dependent autophagy is mainly due to its nuclear or cytosolic function. We seek to define this question using a few different approaches. Fractionation of PC3 cell lysate following knockdown of FoxO3a revealed a significant elevation of cytosolic FoxO1, while the quantity of nuclear FoxO1 remain unchanged (Fig. 5A). This result suggests that the increased FoxO1 resulting from FoxO3a suppression mostly accumulates in the cytosol. We speculate that this predominantly cytosolic elevation of FoxO1 was the likely culprit for the activation of autophagy following FoxO3 knockdown, consistent with some recent reports [33], [34]. Consistent with the fractionation study, RT-PCR quantitation of known FoxO target genes involved in autophagy, such as Atg4c, Atg7, LC3, Atg12, and Bnip3, showed no significant increase in expression upon FoxO3a knockdown even though FoxO1 was elevated as expected (Fig. 5B).

To directly assess the impact of cytosolic FoxO1 on autophagy in PC3 cancer cells, we introduced into cells an expression vector containing Flag-tagged FoxO1 modified to have a defective DNA binding mutation, FoxO1-ΔDB [33]. The FoxO1-ΔDB thus expressed is non-functional as a transcription factor and, interestingly, almost exclusively localized to the cytosol. In both PC3 cells (Fig. 5C) and H1299 cells (Fig. 5D), Flag-FoxO1-ΔDB protein was exclusively localized to the cytosol as expected [33] and Flag-FoxO1-ΔDB expressing cells (red) had markedly higher level of autophagosomes than that of the un-transfected cells. Image analysis has demonstrated statistically significant elevation of autophagosome in cells expressing cytosolically localized FoxO1. These data provide direct evidence to support the notion that the cytosolic accumulation of FoxO1 in these cancer cells indeed promotes autophagy, independent of its nuclear function.

### Cytosolic FoxO1-mediated induction of autophagy is independent of suppression of mTORC1 activity

**Figure 6:**
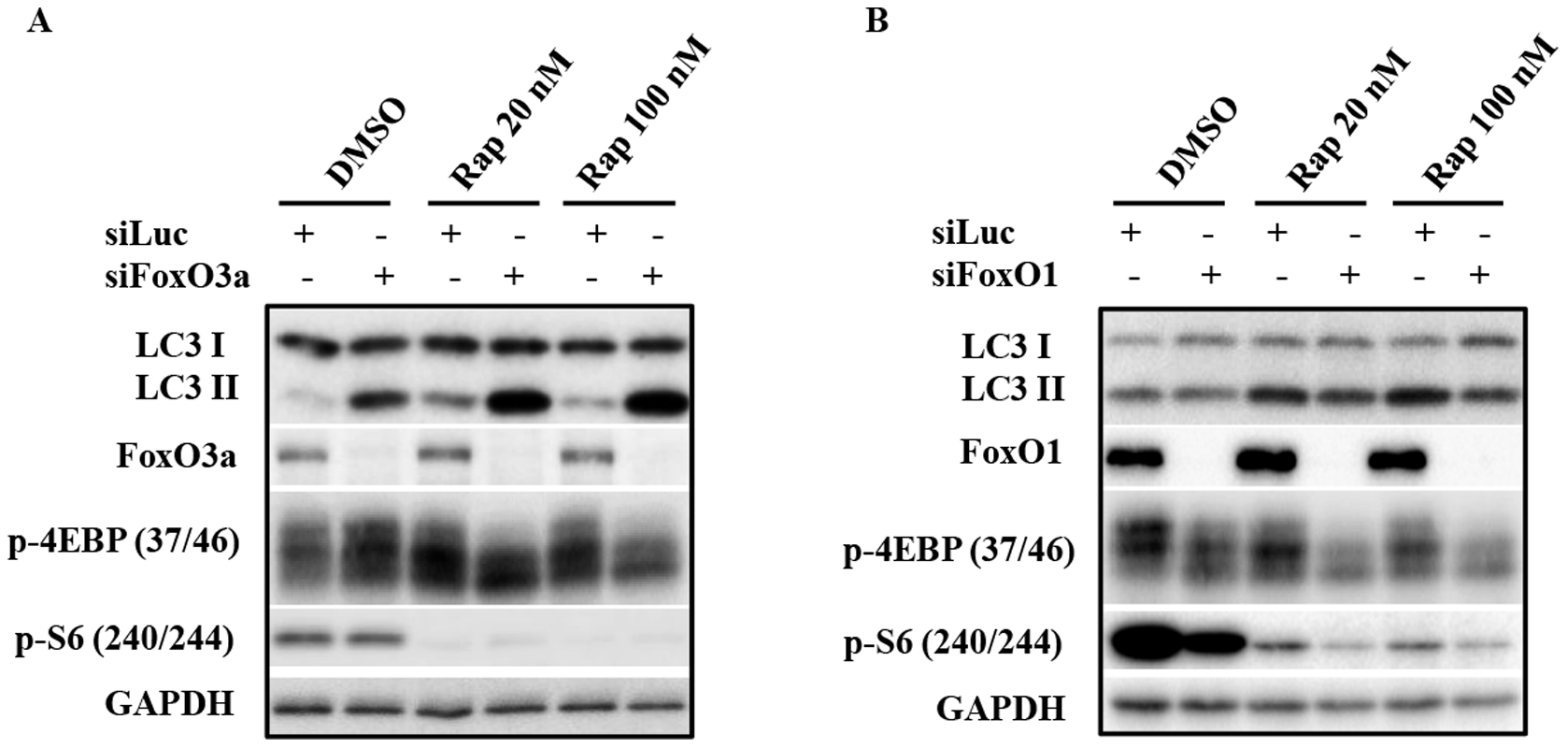
FoxO3a and FoxO1 regulation of autophagy is unlikely to be dependent on mTORC1 activity. (A) PC3 cells were transfected with siRNAs targeting FoxO3a as indicated. Following 48 h of transfection, cells were subjected to rapamycin treatment for 24 h prior to processing for immunoblot analysis of the indicated proteins. (B) PC3 cells underwent similar study as described in (A), except siRNA targeting FoxO1 was used as indicated. All experiments have been performed three times with similar results.

mTORC1 is a well-recognized sensor for nutrition and growth factor signaling; it inhibits autophagy in response to growth signaling. Hence, we examined the activity of mTORC1 signaling when FoxO1 or FoxO3a expression is suppressed with siRNA. FoxO3a knockdown led to a significant elevation of autophagy as seen above; no significant changes in pattern of phospho-4EBP1 and phospho-S6 were observed in cells with FoxO3a knockdown while autophagy was markedly induced (Fig. 6A, DMSO), suggesting that the elevation of autophagy induced by FoxO3a knockdown was likely not through inhibition of mTORC1. Worth noting, FoxO1 knockdown-mediated down regulation of autophagy was accompanied by an inhibition of mTORC1 signaling, based on both phospho-4EBP1 and phospho-S6 patterns (Fig. 6B, DMSO), which also supported the notion that FoxO regulation of autophagy was not mediated through the classic mTORC1 signaling in this case.

To further evaluate the interaction between mTOR signaling and FoxO-regulated autophagy, we combined either FoxO3a or FoxO1 knockdown with rapamycin treatment in PC3 cells. Rapamycin treatment alone effectively blocked mTORC1 function, but induced autophagy to a much less extent than that induced by FoxO3a knockdown. The combination of FoxO3a knockdown and rapamycin treatment resulted in a higher level of autophagy induction (Fig. 6A, Rapamycin). Since the results detailed above indicated that the autophagy regulatory effect of FoxO3a was mediated through FoxO1, we assessed the impact of FoxO1 suppression in combination with rapamycin on mTOR signaling. Consistent with our above results, suppression of FoxO1 inhibited both basal and rapamycin-induced autophagy. Paradoxically, FoxO1 down-regulation appeared to inhibit mTOR function, again supporting the notion that FoxO1 positively regulates autophagy through a different mechanism from that resulting from mTORC1 inhibition (Fig. 6B, Rapamycin). These observations provide compelling evidence that the regulation of autophagy by FoxO proteins is not through the classic manipulation of mTORC1 signaling.

## Discussion

Macroautophagy, commonly referred to as autophagy, has received increasing attention in both basic mechanistic studies as well as for its involvement in multiple pathophysiological processes, including cancer and metabolic disorders. The regulation of autophagy reflects a cells' response to its extracellular environment, such as nutrient, growth factors, cytokines, oxygen status, etc. Indeed, autophagy is downstream of multiple critical signaling pathways, including PI3K/AKT/mTOR and several others that are either parallel to or interactive with this main axis. A number of therapeutic applications are under investigation for either inhibiting or inducing autophagy, depending on the cell context, to treat human diseases, which underscores the importance of thorough understanding of this process [8]. PI3Kinase signaling is central to cellular response to extracellular environment, and a group of the most notable and evolutionarily conserved effectors of this pathway are FoxO proteins, which relay signals to different yet connected downstream components to provide integrated cellular responses to environment [40]. While earlier studies have implied that different members of the FoxO protein family function similarly in promoting autophagy, there has been little investigation on the potential interplay between major family members such as FoxO1 and FoxO3a in different cellular contexts. In this study, we present convincing evidence that FoxO3a can function as a negative regulator of autophagy in cancer cells, including PC3 prostate, MDA-MB-231 breast and HCT116 colon cancer cell lines (Fig. 2). The evidence includes: (i) multiple siRNAs targeting FoxO3a elicit the autophagy induction, ruling out off-target effects, (ii) FoxO3a suppression increased autophagy flux as demonstrated by multiple approaches to examine autophagosome to autophagolysosome progression, (iii) autophagy induction by FoxO3 knockdown could be rescued by ectopic expression of siRNA resistant wild type FoxO3a (Fig. 3), and (iv) quantitation of FoxO1 mRNA demonstrated the effect of FoxO3a gain- and loss-of function on endogenous FoxO1 expression (Fig. 4E).

As noted in the Introduction, a recent study has shown a functional interaction between FoxO3a and FoxO1. In that study, FoxO3a promoted cytosolic localization of FoxO1 by transcriptionally elevating expression of the PI3-kinase catalytic subunit in human embryonic kidney cells and mouse embryonic fibroblast cells [34]; hence FoxO3a acted as a positive regulator of autophagy in those cell types. In regard to the possible mechanism for the difference of FoxO3a regulating FoxO1 and autophagy in the two studies, it seems likely that the differing effects could be due to the cell context differences. In the study by Zhou et al, immortalized benign HEK293T and MEF cells were employed, while the current study uses multiple human cancer cells. These studies highlight that the regulatory impacts of FoxO proteins can be different between different cell types.

In recent years, it has become increasingly recognized that autophagy dysregulation can be part of the molecular pathology in multiple human diseases. Hence, the identification of positive and negative regulators is important in understanding the signaling mechanisms involved in autophagy and its role as adaptive response to various physiological and pathological stresses. In the recent development of therapeutics targeting autophagy, both stimulation and inhibition of autophagy have been reported to be effective approaches [41]–[44], depending on cell signaling context and specific pathology. These findings underscore the importance of a more thorough understanding of the regulatory mechanisms of this process. The new information on FoxO regulation of autophagy advances the understanding of autophagy regulation. Further investigation of the interplay between FoxO3a and FoxO1 in benign and cancer cells, and among different cancer cells will not only advance the understanding of autophagy regulation but may also provide information for targeting autophagy in therapy.
